# The Impact of Varying Food Availability on Gene Expression in the Liver: Testing the Match-Mismatch Hypothesis

**DOI:** 10.3389/fnut.2022.910762

**Published:** 2022-07-01

**Authors:** Janina Feige-Diller, Marisol Herrera-Rivero, Anika Witten, Monika Stoll, Sylvia Kaiser, S. Helene Richter, Norbert Sachser

**Affiliations:** ^1^Department of Behavioral Biology, University of Münster, Münster, Germany; ^2^DFG RTG EvoPAD, WWU Münster, Münster, Germany; ^3^Department of Genetic Epidemiology, Institute of Human Genetics, University of Münster, Münster, Germany; ^4^Core Facility Genomics, Medical Faculty, University of Münster, Münster, Germany

**Keywords:** Match-Mismatch hypothesis, RNA-sequencing (RNA-seq), food availability changes, differential expression (DE) analysis, liver transcriptome, metabolism, immunity and inflammation

## Abstract

**Background:**

During early phases of life, such as prenatal or early postnatal development and adolescence, an organism's phenotype can be shaped by the environmental conditions it experiences. According to the Match-Mismatch hypothesis (MMH), changes to this environment during later life stages can result in a mismatch between the individual's adaptations and the prevailing environmental conditions. Thus, negative consequences in welfare and health can occur. We aimed to test the MMH in the context of food availability, assuming adolescence as a sensitive period of adaptation.

**Methods:**

We have previously reported a study of the physiological and behavioral effects of match and mismatch conditions of high (*ad libitum*) and low (90% of *ad libitum* intake) food availability from adolescence to early adulthood in female C57BL/6J mice (*n* = 62). Here, we performed RNA-sequencing of the livers of a subset of these animals (*n* = 16) to test the effects of match and mismatch feeding conditions on the liver transcriptome.

**Results:**

In general, we found no effect of the match-mismatch situations. Contrarily, the amount of food available during early adulthood (low vs. high) drove the differences we observed in final body weight and gene expression in the liver, regardless of the amount of food available to the animals during adolescence. Many of the differentially expressed genes and the corresponding biological processes found to be overrepresented overlapped, implicating common changes in various domains. These included metabolism, homeostasis, cellular responses to diverse stimuli, transport of bile acids and other molecules, cell differentiation, major urinary proteins, and immunity and inflammation.

**Conclusions:**

Our previous and present observations found no support for the MMH in the context of low vs high food availability from adolescence to early adulthood in female C57BL/6J mice. However, even small differences of approximately 10% in food availability during early adulthood resulted in physiological and molecular changes with potential beneficial implications for metabolic diseases.

## Background

Phenotypic plasticity allows the shaping of an individual's phenotype by the environment it experiences during early phases of life (1). Therefore, the developmental course of an individual might be adjusted according to predictions of the future environmental conditions based on information gained throughout these early phases (1, 2). As stated by the Match-Mismatch hypothesis (MMH), an individual thus effectively matched to the prevailing and future environmental conditions will be less prone to illnesses later in life. In contrast, if the environment drastically changes during the mature phase, a mismatch of the individual's phenotype and the environmental conditions can occur, possibly causing an increased disease susceptibility (3–7). The consequences of such a mismatch might be especially severe if fundamental needs, such as nutrition, are affected. Evidence in humans indicates a mismatch effect caused by discrepancies in food availability between prenatal or early postnatal life and adulthood. Data from historical events, e.g., the Dutch potato famine, regarding a mismatching situation of low food availability during early life and high food availability during later life indicated an increased risk of various cardiovascular and metabolic diseases (8–12). Similarly, individuals shaped by high food availability during early life may have an increased risk to suffer from malnutrition and comorbidities in situations of scarce nutrition, such as prison camps or famines (1, 13). Contrarily, individuals who experienced a matched low food availability were found to have smaller stature and a metabolism that favors the laying down of fat (3, 6), causing a reduced risk to suffer when exposed to poor nutrition (14). Furthermore, in the opposite situation of matched high food availability, a decreased risk of obesity and comorbidities during adulthood has been reported (15–17).

So far, match-mismatch effects have been mainly reported for discrepancies between the prenatal or neonatal phase and later life (9, 14–16, 18, 19). However, adolescence also represents a sensitive developmental period (20, 21) as it is, for example, associated with a modulation of the hypothalamic-pituitary-adrenal (HPA) axis (22), which plays an important role in energy regulation (23). Therefore, we hypothesized that adolescence represents a sensitive period for the long-term shaping of an individual's metabolism. To investigate this, our group applied a match-mismatch design on female mice exposed to low or high food availability in adolescence and early adulthood. Recently, we reported that the match-mismatch situation showed no significant effects on the welfare and health of the animals, as assessed by physiological and behavioral measurements. However, various effects of low vs. high food availability were observed, including lower relative liver weights and glucocorticoid secretion in a situation of plentiful nutrition. Taken together, the effects suggested a short-term adjustment of the metabolism and immune system to the prevailing situation of food availability (24). Here, we used RNA-sequencing to investigate changes in the liver transcriptome of a subset of these animals exposed to matching or mismatching food availability in adolescence and early adulthood. The liver is fundamentally important for lipid and glucose metabolisms [reviewed in Rui (25)], but also strongly involved in immune function (26–28). Consistent with our previous findings, we found no match-mismatch effects. Transcriptional changes in the liver were rather induced by low vs. high food availability during early adulthood, and implicated a wide range of biological processes related to metabolism as well as, to some extent, to immunity and inflammation.

## Methods

### Experimental Animals and Collection of Tissue Samples

**Figure 1:**
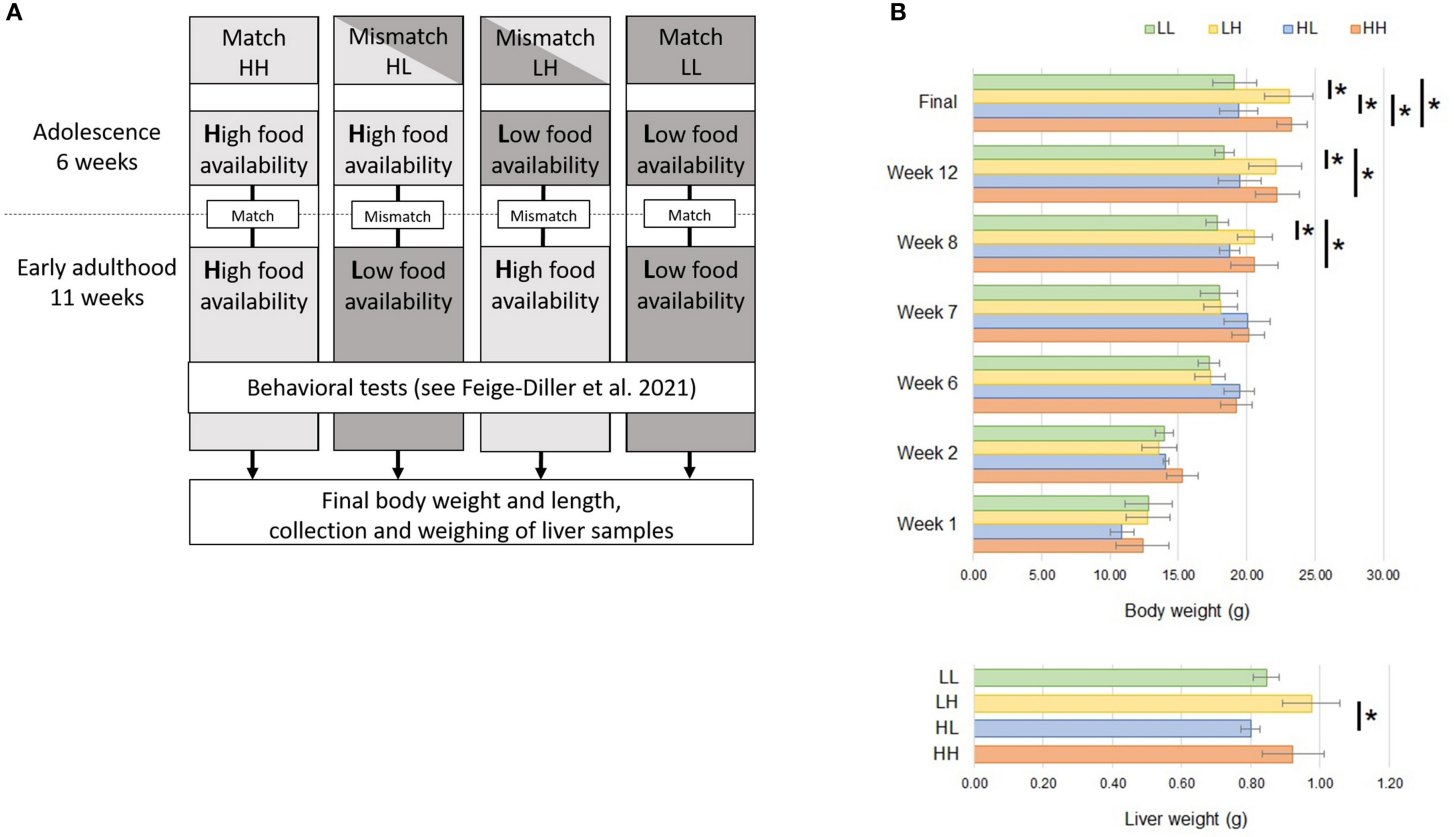
**(A)** Experimental design. Mice were kept at high (H) or low (L) food availability conditions during 6 weeks of adolescence. Upon reaching early adulthood, food availability either remained the same (match) or was changed (mismatch) for another 11 weeks. During the last 5 weeks, behavioral tests were conducted for a previous publication (24). Subsequently, final body weights and body length were assessed; liver samples were weighed and collected for RNA-sequencing. **(B)** Progressive increases in body weight were assessed at weeks 1, 2, 6, 7, 8, 12 and at the end of the experiment. Afterwards, liver weight was recorded. Significant differences (Tukey HSD adj. *p* < 0.05) between groups are denoted by (*). L, low food availability; H, high food availability.

All mice used for the present study were part of our previously published work, and detailed experimental protocols have been described (24). Briefly, female C57BL/6J mice were assigned to matching or mismatching conditions of food availability during adolescence and early adulthood to test the Match-Mismatch hypothesis (Figure 1A). High food availability was simulated by *ad libitum* access to a standard laboratory food (Altromin 1324, Altromin GmbH, Lage, Germany). In contrast, low food availability was characterized by feeding the animals once per day with a mildly restricted diet (10% reduction of the *ad libitum* intake). In phase 1, two groups of adolescent animals (postnatal day-PND- 28–70 ± 1) were exposed to low (L) food availability, while another two groups were exposed to high (H) food availability for a period of 6 weeks. During this phase, animals were not considered fully-grown. L and H cages were paired according to matching body weights. The amount of food in the L cage was then kept to approximately 90% of its respective H cage. In this manner, the continued growth of animals in the L cages, despite of food restriction, was warranted (29, 30). In phase two, when animals were considered to have reached early adulthood and full growth (PND 71 ± 2), they were kept either in the same (match) or the opposite (mismatch) situation of food availability for another 6 weeks. During this phase, animals exposed to low food availability were either kept to 90% of their own maximum body weight (group HL) or to that of the mice in the respective paired cage (group LL) during the last week of the previous phase. The groups studied thus consisted of the match or mismatch conditions HH (*n* = 15), HL (*n* = 15), LH (*n* = 16) and LL (*n* = 16). Subsequently, all animals underwent a series of behavioral tests over a period of 5 weeks (PND 111 – 142 ± 1), while remaining in the same situation as after the match/mismatch. At least 5 days after the last behavioral test, 16 randomly selected animals were anesthetized using 2.5% isoflurane in oxygen and decapitated. After dissection and removal of excessive fat tissue, the weights of the hearts, kidneys, livers, spleens and adrenal glands were measured. Livers were snap-frozen in liquid nitrogen and stored at −70°C.

All experimental procedures complied with the regulations covering animal experimentation within Germany (Animal Welfare Act) and the EU (European Communities Council DIRECTIVE 2010/63/EU). The study was approved by the corresponding local (Gesundheits-und Veterinäramt Münster, Nordrhein-Westfalen) and federal authorities (Landesamt für Natur, Umwelt und Verbraucherschutz Nordrhein-Westfalen ‘LANUV NRW,” reference number 84–02.04.2018.A067).

### Statistical Testing of Descriptive Phenotypes

Differences between the match and mismatch groups in terms of body weight at weeks 1, 2, 6, 7, 8, and 12, final body weight, final body length, weight of the liver and relative weight of the liver were tested through analysis of variance (ANOVA), followed by the Tukey's honest significant difference (HSD) method applied with a family-wise confidence level of 95%. Significance was set at *p* < 0.05.

### RNA Extraction, Library Preparation and Sequencing

Liver tissue samples from four animals in each match or mismatch group (HH, LL, HL and LH) were destined for analysis of their transcriptomes via RNA-sequencing (RNA-seq). Total RNA was isolated using the Direct-zol RNA Microprep Kit (Zymo Research), followed by a DNase digestion step. Library preparation was carried out upon mRNA enrichment with the NEBNext Poly(A) mRNA Magnetic Isolation Module, using the NEBNext Ultra II RNA Directional Library Prep Kit for Illumina (New England BioLabs). Single read sequencing took place on a NextSeq 500 System (Illumina), using the corresponding NextSeq 500 High Output Kit v2.5, with a read length of 75 base pairs. The integrity of the RNA and quality of the library were assessed using a TapeStation 4200 (Agilent).

### Data Pre-processing

Using a molecular barcode, the data was automatically demultiplexed using the Illumina bcl2fastq2 Conversion Software v2.20. FastQ files underwent two rounds of quality control, pre-trimming and post-alignment, using FastQC v0.11.7 (31). Removal of Illumina adapters and low-quality sequences was performed with Trimmomatic v0.38 (32). Reads of length<15 bases, as well as leading and/or trailing bases with quality<3 or no base call, and bases with average quality<15 in a 4-base sliding window were removed. Alignment was performed with HISAT2 v2.1.0 (33) using the mouse genome assembly mm10 (*Mus musculus*, GRCm38). Mapped reads (primary alignments) were sorted by read name using SAMtools v1.8 (34), and read counts were calculated with HTSeq v0.11.2 (35).

### Differential Expression and Functional Enrichment Analyses

Differential expression between all possible combinations of match or mismatch groups (HH vs. HL, HH vs. LH, HL vs. LH, LL vs. HH, LL vs. HL and LL vs. LH) was assessed using DESeq2 (36). Raw read counts were filtered to remove genes with <10 counts prior to analysis. DESeq2 tests for the statistical significance of coefficients in a negative binomial generalized linear model using the Wald test, and corrects the *p*-values for multiple comparisons according to the Benjamini-Hochberg method. These models were adjusted for experimental batch of the animals. Genes were considered differentially expressed when adjusted-p (p.adj) <0.05 (a log2 fold change (lfc) threshold was not applied). Furthermore, to provide a biological context to these findings, each list of differentially expressed genes (DEGs) was subjected to functional enrichment analysis for Gene Ontology biological processes (GO_BP) in *Mus musculus* using BiNGO v3.0.3 (37) for Cytoscape v3.7.1 (38). GO_BP terms were considered significantly enriched following a hypergeometric test for overrepresentation, corrected for multiple comparisons according to the Benjamini-Hochberg method (p.adj<0.05).

## Results

**Figure 2:**
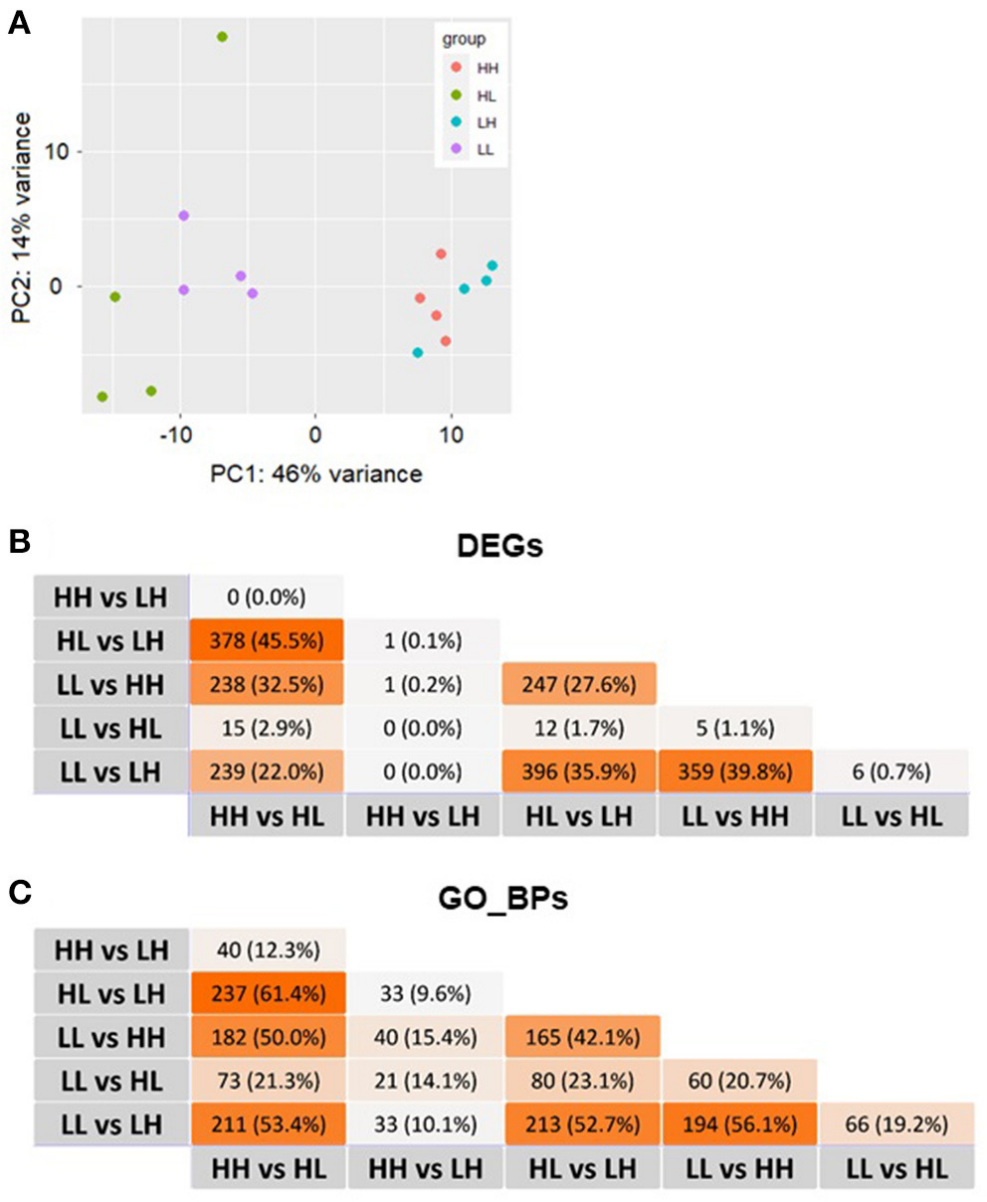
**(A)** Plot of the first 2 principal components (PCs) of the normalized read counts. **(B)** Overlaps of differentially expressed genes (DEGs) and **(C)** the corresponding enriched Gene Ontology biological processes (GO_BPs) among all comparisons. No DEGs and/or GO_BP terms overlapping among all comparisons performed were identified. L, low food availability; H, high food availability.

A basic description of the subsample used for transcriptomic profiling of the livers of mice placed in match or mismatch combinations of low and high food availability from our previous report (24) is shown in Table 1. In this subsample of 16 mice, we observed differences in the animals' body weights from week 8, when mice in the LH and HH groups started to show significant weight gains (week 8 *p* = 0.0165, week 12 *p* = 0.0077, final measurement *p* = 0.0017) in comparison to animals subjected to the LL and HL conditions. Liver weights differed only between mismatch groups, as the higher and lower values were found for these mice. Livers of animals in the LH group were significantly heavier than those in the HL group (Figure 1B). From RNA-seq, alignment rates > 97% and over 25 million reads were obtained for all samples. Visualization of the first two principal components (PCs) of the normalized read counts showed clustering of the liver transcriptomes according to food availability during phase two, which could explain up to 46% of the phenotypic variance between all samples (Figure 2A).

**Table 1:** Basic description of the sample used for transcriptomic profiling by RNA-sequencing.

	**HH**		**HL**		**LH**		**LL**		***P*-val**	**Pairwise** **significant**
	***Mean***	***SD***	***Mean***	***SD***	***Mean***	***SD***	***Mean***	***SD***		
Samples	4		4		4		4			
Week 1 (g)	12.38	1.75	10.88	1.61	12.78	0.86	12.83	1.90	0.3080	
Week 2 (g)	15.28	0.67	14.08	1.26	13.60	0.22	13.95	1.15	0.1090	
Week 6 (g)	19.23	0.79	19.45	1.13	17.33	1.13	17.23	1.16	0.0173	
Week 7 (g)	20.13	1.33	20.03	1.21	18.08	1.69	17.98	1.18	0.0725	
Week 8 (g)	20.55	0.83	18.75	1.29	20.58	0.76	17.83	1.70	0.0165	LL vs. HH *p* = 0.0342; LL vs. LH *p* = 0.0325
Week 12 (g)	22.23	0.71	19.46	1.93	22.10	1.57	18.35	1.59	0.0077	LL vs. HH *p* = 0.0164; LL vs. LH *p* = 0.0202
Final body weight (g)	23.31	1.61	19.40	1.74	23.07	1.40	19.09	1.11	0.0017	HL vs. HH *p* = 0.0132; LL vs. HH *p* = 0.0787; LH vs. HL *p* = 0.0199; LL vs. LH *p* = 0.0118
Liver weight (g)	0.92	0.09	0.80	0.03	0.98	0.08	0.85	0.04	0.0112	LH vs. HL *p* = 0.0117
Rel. weight-liver (%)	3.96	0.34	4.16	0.51	4.23	0.20	4.44	0.37	0.3710	
Final body length (cm)	9.43	0.26	9.00	0.42	9.40	0.36	9.08	0.67	0.4560	

*L, low food availability; H, high food availability; SD, standard deviation*.

*P-val, p-value from ANOVA test, pairwise significant: adjusted p-value from Tukey post-hoc test*.

**Figure 3:**
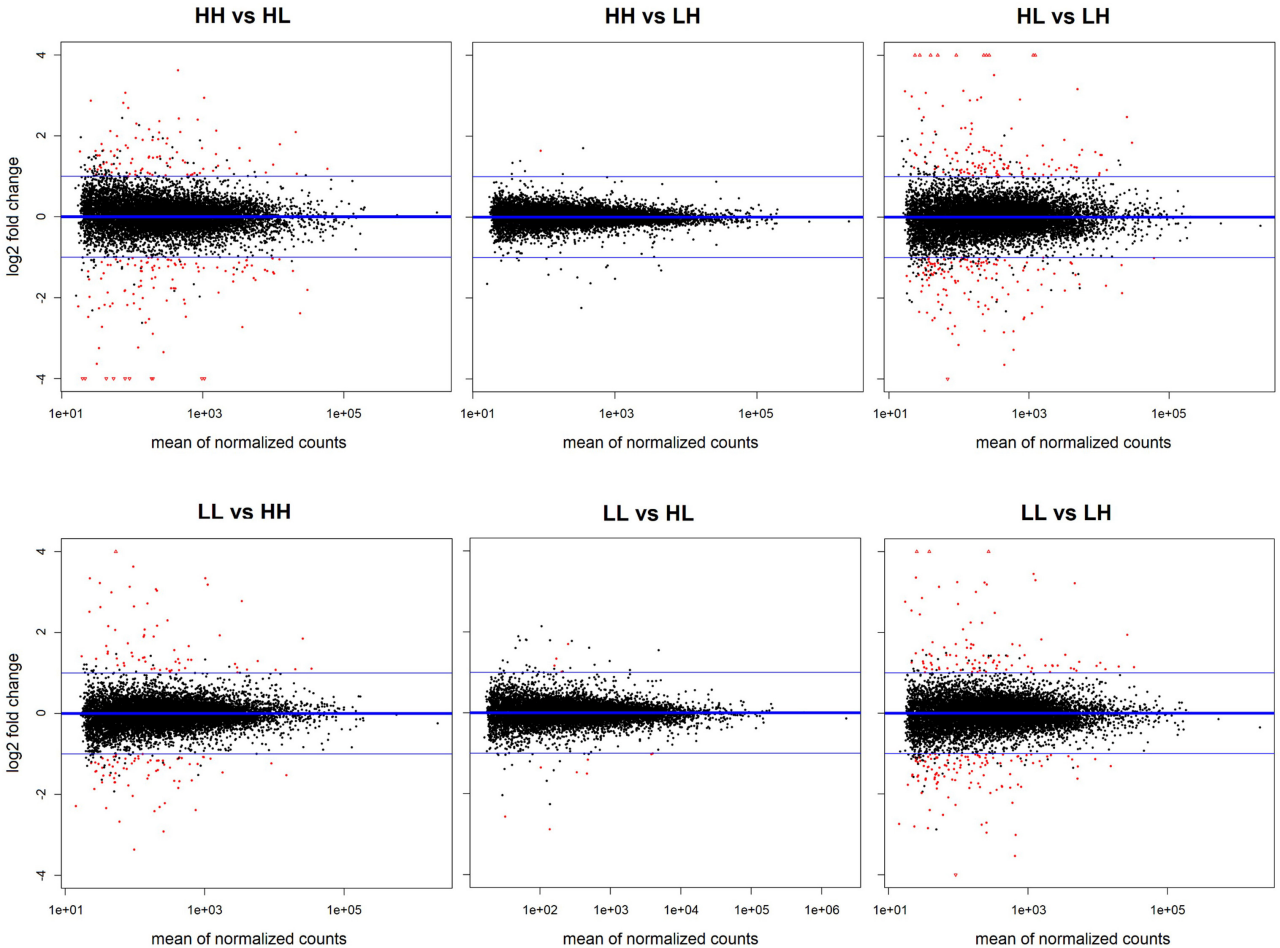
Overview of differential expression results for all comparisons. MA plots show the statistically significant (adjusted *p* < 0.05) dysregulations obtained in each comparison marked in red.

The results obtained from the series of differential expression (Figure 3, Supplementary Tables 1–6) and corresponding functional enrichment analyses performed (Supplementary Table 7) are summarized in Table 2. In general, judging by the numbers of DEGs found for each comparison and, similar to what was observed for the final body weight, the liver transcriptomes changed significantly between match or mismatch groups with opposing feeding conditions during early adulthood. This suggested that the observed molecular changes were induced by the later (early adulthood) rather than by the earlier (adolescence) diet scheme, and that there was no significant effect of the match-mismatch situation in our study. Moreover, many overlaps were observed among DEGs (Figure 2B) and GO_BP terms (Figure 2C) between comparisons. When food availability was the same during early adulthood, changes in the liver transcriptomes between animals were limited. Nevertheless, a higher proportion of the GO_BP terms enriched in the latter case were specific for these comparisons. Table 3 provides a summary of the comparison-specific GO_BP terms found for each group comparison performed.

**Table 2:** Summary of results from the differential expression and functional enrichment analyses.

**Comparison**	**Type**	**# DEGs**	**# Up**	**# Down**	**# GO_BPs**	**# Specific GO_BPs (%)**
LL vs. HH	M vs. M	452	221	231	291	37 (12.7)
HH vs. HL	M vs. MM	518	227	291	459	85 (18.5)
HH vs. LH	M vs. MM	3	1	2	40	19 (47.5)
LL vs. LH	M vs. MM	808	397	411	426	86 (20.2)
LL vs. HL	M vs. MM	19	8	11	104	43 (41.3)
HL vs. LH	MM vs. MM	691	391	300	467	86 (18.4)

*L, low food availability; H, high food availability; M, match; MM, mismatch; DEGs, differentially expressed genes; Up, upregulated; downregulated; GO_BPs, Gene ontology biological processes*.

**Table 3:** Summary of biological processes enriched for each statistical comparison in a specific fashion.

**Comparison**	**Specific gene ontology biological process terms (examples/categories)**
HH vs. HL	Antigen processing and presentation; calcium ion homeostasis; cell communication; response to metals, insulin, peptide hormones; enteric nervous system development; mating; myelination; regulation of cytoskeleton organization, innate immune response, ubiquitination, sodium transport, tissue remodeling, cell migration; proteolysis; white fat cell differentiation.
HH vs. LH	Appendage development, cell fate determination, zinc ion homeostasis, response to copper ion, meiosis.
HL vs. LH	Apoptosis, cytolysis; bone remodeling; Wnt signaling; carnitine, ceramide, mannose metabolism; cation transport; cell activation involved in immune response, T cell differentiation; erythrocyte development; fatty acid beta oxidation; regulation of inflammatory response, hormone secretion, striated muscle contraction, signaling, cholesterol storage.
LL vs. HH	Androgen metabolism, embryonic development, heart contraction, macromolecular complex assembly, lipoprotein particle remodeling.
LL vs. HL	Cellular responses to fatty acids, heparin, lipids; biosynthesis of cytokines, triglycerides; secretion of insulin and peptides; regulation of heart rate, histone modifications.
LL vs. LH	Carbohydrate homeostasis; response to carbohydrate stimulus, nutrient levels; chitin and energy reserve metabolism; cellular localization; glycosylation; mitochondrion organization; regulation of cell adhesion, transcription, arterial blood pressure; renal system; respiratory tube and salivary gland development.

In more detail, differential expression analysis between the match-mismatch groups LL and LH showed the largest number of DEGs (808; Supplementary Table 6), with similar numbers of these being up- (397) or down-regulated (411). The top 5 DEGs were: *sult2a5* (p.adj = 2.76 x 10^−39^, lfc = −3.01), *plin4* (p.adj = 1.44 x 10^−34^, lfc = −2.96), *mup3* (p.adj = 1.91 x 10^−33^, lfc = 1.94), *sult2a3* (p.adj = 6.91 x 10^−31^, lfc = −3.53) and *gm10804* (p.adj = 2.07 x 10^−22^, lfc = −4.75). A great proportion of DEGs and enriched GO_BP terms were not specific for this comparison, where a particularly high overlap with the comparison between match groups (LL vs. HH; about 40% in DEGs and 56% in GO_BPs) was found (Figures 2B,C). Specific functional enrichments for the comparison LL vs. LH accounted for about 20% of the enriched terms (86/426) and included GO_BP terms related to the cardiovascular and respiratory systems, as well as to metabolism, such as carbohydrate homeostasis and responses to nutrient levels, and to cellular localization, cell adhesion and glycosylation, among others (Supplementary Table 7). In contrast, the analyses comparing HH vs. LH and LL vs. HL found only three (*rap1gap*: p.adj = 0.011, lfc = −0.88; *cyp26b1*: p.adj = 0.017, lfc = 1.64; and *mt1*: p.adj = 0.018, lfc = −0.98) and 19 DEGs, respectively (Supplementary Tables 2, 5). Between LL and HL, the top 5 DEGs identified were: *sult2a7* (p.adj = 3.88 x 10^−5^, lfc = −2.88), *gadd45g* (p.adj = 5.13 x 10^−4^, lfc = 1.34), *1810008I18Rik* (p.adj = 1.17 x 10^−3^, lfc = −0.83), *srebf1* (p.adj = 1.27 x 10^−3^, lfc = −0.79) and *lrtm1* (p.adj = 2.28x10^−3^, lfc = −1.16). Because in these cases we allowed functional enrichments to take place with only one overlapping gene, however, there were large numbers of significantly overrepresented GO_BP terms (40 and 104, respectively). From these, 47.5% were specific for the HH vs. LH comparison, while 41.3% were specific for the LL vs. HL comparison (Supplementary Table 7). Nevertheless, these latter enrichment results should be interpreted with caution.

Moreover, analysis of both match groups (LL vs. HH) resulted in 452 DEGs and 291 overrepresented GO_BP terms (Supplementary Tables 4, 7). The top 5 DEGs were: *pde6c* (p.adj = 2.24 x 10^−27^, lfc = −2.22), *mup17* (p.adj = 2.03 x 10^−25^, lfc = 3.18), *mup3* (p.adj = 5.67 x 10^−25^, lfc = 1.85), *mup6* (p.adj = 2.61 x 10^−23^, lfc = 2.17) and *plin4* (p.adj = 2.33 x 10^−21^, lfc = −2.92). From the GO_BP terms, 37 (12.7%) were comparison-specific and included terms such as androgen metabolism, embryonic development, heart contraction, and lipoprotein particle remodeling, among others. On the other hand, comparing both mismatch groups (HL vs. LH) resulted in 691 DEGs and 467 GO_BP terms (Supplementary Tables 3, 7), showing a particularly important overlap with the analysis of HH vs. HL (45.5% in DEGs and 61.4% in GO_BPs; Figures 2B,C). This overlap was even found within the top 5 DEGs in these comparisons with the gene *mup7* (HL vs. LH: p.adj = 2.04 x 10^−29^, lfc = 5.03; HH vs. HL: p.adj = 4.98 x 10^−19^, lfc = −4.86). For the HL vs. LH comparison, 86 (18.4%) of the overrepresented GO_BP terms were specific and included terms related to metabolism, cell signaling and cell death, immunity, beta-oxidation and cholesterol storage, among others (Supplementary Table 7).

**Figure 4:**
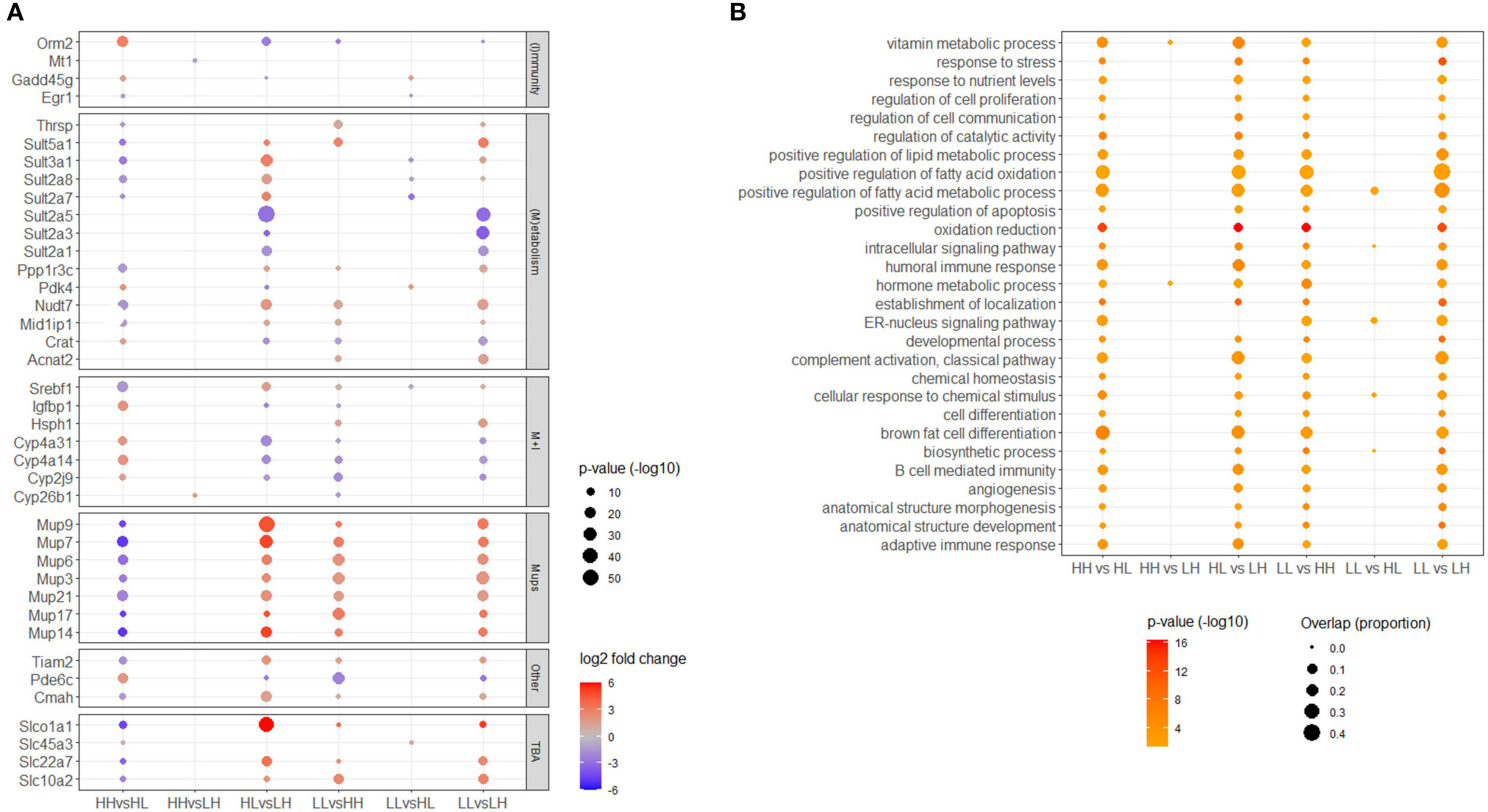
**(A)** Top genes. Selection was based on the gene being differentially expressed within the top 20 most significant hits in more than one comparison, and belonging to a recognizable cluster of functional categories. TBA: transport of bile acids. **(B)** Representative top shared Gene Ontology biological processes (GO_BPs). Selected terms are representative of all different GO_BP terms enriched in at least four of the comparisons. Only significant (adjusted *p* < 0.05) genes and functional terms are shown.

As mentioned above, many overlaps among comparisons were identified for both, DEGs and enriched GO_BP terms (Figures 2B,C). In the case of DEGs, at least four distinctive functional categories common to all comparisons were recognized among a maximum of 20 of the most significant genes obtained from each differential expression analysis. These categories were metabolism, immunity, transport of bile acids and major urinary proteins (Mups). Together, top DEGs resulting from our comparisons highlighted the importance of 64 genes in the transcriptional responses to patterns of food availability in the mouse liver. These included genes coding for various sulfotransferases (e.g. *sult2a5, sult3a1, sult5a1*), cytochrome P450 proteins (e.g. *cyp4a14, cyp2j9*), solute carriers (e.g. *slc22a7, slc10a2, slco1a1*), Mups (e.g. *mup9, mup14, mup21*), and regulatory molecules, including metabolic (e.g. *mid1ip1, ppp1r3c, rarres1*), immune (e.g. *dntt, egr1, gadd45g*) and circadian (e.g. *dbp*) regulators (Figure 4A). Furthermore, in the case of overrepresented GO_BPs, 156 terms were found commonly enriched in a maximum of four to five of the performed comparisons (Supplementary Table 7). These terms could be grouped into the following broad functional categories: metabolism, homeostasis, immunity, differentiation, cellular responses, localization, molecular transport, oxidation-reduction and apoptosis/cell death. Some of the terms in these categories have been manually selected to represent these top shared GO_BPs and are shown in Figure 4B. No DEGs and/or GO_BP terms overlapping among all comparisons performed were identified.

## Discussion

According to the MMH, negative effects on health can occur if the environment in which an individual was shaped differs from the environment it later lives in (3–7). Noticeably, match-mismatch studies so far focused on the long-term shaping of the phenotype during prenatal and early postnatal development. Contrarily, adolescence has only recently gained attention as a time period during which shaping of the phenotype in response to the prevailing environmental conditions can occur (20, 21).

To test the MMH assuming adolescence as the sensitive period of developmental phenotypic plasticity, we used female C57BL/6J mice exposed to matching or mismatching situations of low and high food availability in adolescence and early adulthood. Here, we reported our study of differential gene expression in the liver using RNA-seq. In accordance with our previous assessments of physiological and behavioral parameters (24), our results suggested no match-mismatch effects, indicating that no long-term shaping occurred in response to food availability during adolescence. However, relatively large changes in the liver transcriptome were observed according to the amount of food available to the animals during early adulthood. These changes involved mainly a plethora of metabolic pathways and, in a lower extent, immune processes. Similarly, in our previous publication (24), various effects of low vs. high food availability were observed regarding the activation of the HPA axis (39), as well as of organ weights, including liver and spleen. Interestingly, we previously observed increased liver weights in mice of the LL group, compared to mice in the HH group, when the measurements were based on a subsample of 10 animals per group (24) whereas, in the sequencing subsample reported herein, comprised of 4 mice per group, the differences in liver weights were found between the match-mismatch LH and HL conditions, which aligned better with our expectations. Taken together, our results suggested a short-term adjustment of the metabolism and, possibly, the immune system to the prevailing situation of food availability.

In accordance with our findings, an impact on gene expression levels of the currently prevailing food availability has been previously reported. For example, a study showed that prolonged feeding of a high-fat diet to adult mice induced significant differences in the expression of hepatic genes associated with metabolism, immune system and other biological processes. In particular, several genes involved in defense responses against a lipotoxic environment, oxidative stress and inflammatory processes were upregulated (40). Furthermore, it was also shown that feeding a restricted diet (60% of *ad libitum* intake) decreased the expression levels in heart, liver and hypothalamus of genes associated with inflammation (41). This is in line with a chronic elevation of corticosterone release observed previously in mice and rats fed a restrictive diet (24, 42), which is hypothesized to enhance protective mechanisms against stressors (42–44) and is likely to have a profound anti-inflammatory effect (45). Correspondingly, there is compelling evidence for health improvements in rats fed with even mildly restrictive (down to 10% of *ad libitum* intake) diets (46–48). Taken together, the evidence suggests that high food intake is associated with inflammation and activation of the immune system [reviewed in Reilly & Saltiel (49) and Berg & Scherer (50)]. This may lead to conditions linked to chronic inflammation, such as obesity and its comorbidities, and increase the risk of cardiovascular disease [reviewed in Karczewskiet al. (51), Libby (52)]. Indeed, low-grade chronic inflammation is suggested as an underlying cause of the well-established negative health consequences of *ad libitum* feeding in rodents (53–57). Therefore, our study agrees with previous evidence and supports the notion that a high food intake due to unrestricted food availability might act as a risk factor for metabolic disease partially through the activation of inflammatory and immune processes while, contrarily, mild to moderate food restriction might have anti-inflammatory and immunosuppressive effects that favor healthier metabolic outcomes. However, we found no compelling evidence to support long-term metabolic effects of exposure to restricted or unrestricted diets in adolescence nor to the match-mismatch situation, suggesting that the molecular changes we observed herein were driven by acute responses to the current feeding situation.

A more detailed inspection of the top 20 DEGs from all comparisons performed in our study revealed that, beyond metabolism and immunity, other top genes belonged to the Mups and bile acid transporters. Mups are mainly expressed in the liver and secreted into the urine (58). Recently, they have been linked to metabolic diseases, such as obesity and type 2 diabetes (59). In accordance with the present study, decreasing the caloric intake of mice was shown to cause a reduction of expression levels of various Mup genes in the liver (60, 61). Such a decrease of expression levels in a situation of nutritional scarcity might reduce energy expenditure by decreasing protein loss via the urine (58). In contrast, increased expression levels when food is available in abundance might help to alleviate the negative consequences of increased food intake by utilizing unneeded energy for Mup production, as well as increasing locomotor activity and glucose tolerance (62). Moreover, Mups have been associated with social communication via pheromones in the urine [reviewed in Charkoftakiet al. (59)]. While the literature focuses on the effects of male Mups on females [e.g., (63, 64)], the role of female Mup expression on conspecifics and within the producing animal is less clear. Taken together with our previous observation of a change to social interest in an unfamiliar female caused by differences in food availability, the differential expression of Mups possibly indicates a link of the internal Mup expression pattern and the social behavior of females. Moreover, bile acid transporters have recently come into focus in the context of metabolic diseases (65). Bile acids (BA) are synthesized from cholesterol in the liver and secreted into the small intestine, where they are crucial for the absorption of dietary fatty acids, cholesterol and fat-soluble vitamins (66). In addition, they also act as signaling molecules, affecting for example bile acid metabolism, glucose homeostasis and lipid metabolism (67, 68). A disruption of the cholesterol and BA homeostasis is associated with metabolic diseases (67, 68). Bile acid transporters are likely to play an important role in maintaining BA homeostasis, as they are indispensable for the reabsorption of 95% of BA from the intestines (69). Indeed, there is evidence that a disruption of the well characterized ileal apical sodium bile acid cotransporter (*Slc10a2*) can cause an increased fecal loss of BA resulting in increased BA synthesis (69). A high expression level of transporters under high food availability, as observed herein, might lead to increased reuptake of BA from the intestines, in turn inhibiting the synthesis of new BA. This would lead to less absorption of cholesterol and fats, possibly alleviating the negative effects of high food intake (70).

We acknowledge some limitations of our study. Negative effects associated with a mismatch in humans, such as metabolic diseases, occur predominantly in later life stages (7). Therefore, effects on health and welfare caused by a mismatch situation in mice might not yet have manifested during early adulthood. Nonetheless, in case of a lasting effect of food availability during adolescence on late adulthood, first indications could be expected during earlier life stages. Instead, only effects of the currently prevailing food availability during early adulthood were found. Moreover, most evidence for the MMH has been gathered from situations of very low food availability, such as during a famine, compared to food in abundance (8–12, 18, 71). The discrepancies of approximately 10% between high and low food availability in our study might have been too mild to cause any long-term changes to the phenotype during adolescence.

## Conclusions

We hypothesized that adolescence might represent a sensitive period for the long-term metabolic shaping. As such, we expected to observe physiological, behavioral as well as molecular differences in animals that were exposed to different schemes of mildly restrictive (10% for 11 weeks)/unrestrictive food availability during adolescence compared to early adulthood. Nevertheless, results from either our previous physiological and behavioral studies or the current molecular study support a match-mismatch effect between adolescence and early adulthood when a mildly restrictive diet is implemented. Taken together, physiological, behavioral and molecular differences observed in the liver transcriptome between our study groups mainly pointed toward short-term changes in metabolic and immune pathways. These results might have implications for the development of obesity and other metabolic diseases. Further work should explore the possibility of a match-mismatch effect between adolescence and (early) adulthood at higher dietary restrictions and systematically assess whether dietary restriction in adolescence results beneficial for the health status later in life.

## Data Availability Statement

Raw and processed RNA-seq data has been deposited into NCBI's Gene Expression Omnibus (GEO) database and is accessible with the ID GSE200731.

## Ethics Statement

The animal study was reviewed and approved by Gesundheits-und Veterinäramt Münster, Nordrhein-Westfalen and Landesamt für Natur, Umwelt und Verbraucherschutz Nordrhein-Westfalen.

## Author Contributions

MS, SK, SR, and NS conceived the study and designed the experiments. JF-D carried out the experimental procedures and sample collection, and prepared the manuscript. MH-R processed and analyzed the data and prepared the manuscript. AW supervised sequencing. All authors reviewed and approved the final version of the manuscript.

## Funding

This work was supported by a grant from the German Research Foundation (DFG) to NS (281125614/GRK2220, Project B5).

## Conflict of Interest

The authors declare that the research was conducted in the absence of any commercial or financial relationships that could be construed as a potential conflict of interest.

## Publisher's Note

All claims expressed in this article are solely those of the authors and do not necessarily represent those of their affiliated organizations, or those of the publisher, the editors and the reviewers. Any product that may be evaluated in this article, or claim that may be made by its manufacturer, is not guaranteed or endorsed by the publisher.

## Abbreviations

BA: bile acids
DEGs: differentially expressed genes
GO_BP: Gene Ontology biological processes
H: high food availability
HH: high/high food availability match group
HL: high/low food availability mismatch group
L: low food availability
LH: low/high food availability mismatch group
LL: low/low food availability match group
MMH: Match-Mismatch hypothesis
Mups: major urinary proteins
PCs: principal components
PND: postnatal day
RNA: ribonucleic acid
RNA-seq: RNA-sequencing
SD: standard deviation.
